# C-reactive protein testing to guide antibiotic prescribing for COPD exacerbations: A protocol for systematic review and meta-analysis

**DOI:** 10.1097/MD.0000000000021152

**Published:** 2020-07-17

**Authors:** Xing An, Chuantao Zhang, Xiangwen Weng, Wei Xiao, Zengtao Sun, Zhu Zeng, Qingsong Huang

**Affiliations:** a Respiratory Department, Hospital of Chengdu University of Traditional Chinese Medicine; b Clinical Medical College, Chengdu University of Traditional Chinese Medicine; c Department of Critical Medicine, Hospital of Chengdu University of Traditional Chinese Medicine, Chengdu, China.

**Keywords:** acute exacerbation of chronic obstructive pulmonary disease, antibiotic, C-reactive protein, meta-analysis, protocol, systematic review

## Abstract

**Background:**

The use of antibiotics in the acute exacerbations of chronic obstructive pulmonary disease (COPD) remains controversial. Serum C-reactive protein (CRP), a sensitive biomarker for systemic inflammation and tissue damage, is a good indicator of lower respiratory tract bacterial infection. However, due to the small sample size of the existing studies, the clinical value of CRP in guiding the use of acute exacerbation of chronic obstructive pulmonary disease (AECOPD) antibiotics is insufficient. The aim of this study was to evaluate the value of CRP-guided treatment strategies for AECOPD patients.

**Methods:**

This review summarizes and meta-analyses randomized controlled trials (RCTs) of CRP guiding antibiotic prescribing for COPD exacerbations. RCTs compare either usual-care or the GOLD strategy have been included. The following electronic databases have been searched: PubMed, Cochrane Library, Embase, CNKI, CBM, VIP, and Wanfang Data. The methodologic quality of RCTs has been assessed using the Cochrane risk assessment tool. All trials included are analyzed according to the criteria of the Cochrane Handbook. Review Manager 5.3, R-3.5.1 software, and GRADE pro GDT web solution are used for data synthesis and analysis.

**Results:**

This review evaluates the effects of CRP testing on the antibiotic use, CCQ, EQ-5D utility scores and adverse events in patients with COPD exacerbations.

**Conclusion:**

This review provides clear evidence that CRP testing can reduce the use of antibiotics in patients with AECOPD without causing harm.

## 1 Introduction

Chronic obstructive pulmonary disease (COPD) is an airway/lung inflammatory disease characterized by persistent progressive airflow restriction, ranking 4th in global mortality. ^[1]^ COPD patients experienced an average increase of 1.5 times per year, ^[2]^ accompanied by a rapid decline in lung function and a heavy economic burden.^[3]^ Frequency of acute exacerbations were associated with an increased risk of death.^[4]^ Respiratory tract infection (virus or bacteria) is an important factor inducing acute exacerbation of COPD, about 85% of AECOPD is caused by infection, of which about 50% is caused by bacterial infection and 35% is caused by viral infection.^[5]^ Therefore, the use of antibiotics in clinical practice is still controversial. Reducing overprescribing of antibiotics for respiratory tract infections is essential in an era of increasing antimicrobial resistance.

The guidelines of the global initiative for chronic obstructive lung disease recommend the use of antibiotics in moderately or severely ill patients with acute exacerbations of COPD who have increased cough and sputum purulence. Recommendations for antibiotic prescribing in primary care practice are generally based on clinical features alone (e.g., the Anthonisen criteria^[6]^). However, these characteristics are subjective, and are not accurate enough to predict which patients can be safely treated without antibiotics.^[7]^ Therefore, the definition of a biomarker, which potentially detects such episodes or specific to one subtype of exacerbation would be of great interest.

Serum C-reactive protein (CRP), a kind of acute phase protein, is a sensitive biomarker of systemic inflammation and tissue damage, and is not affected by anti-inflammatory drugs, immunosuppressants, hormones,^[8]^ which can better reflect the bacterial infection of lower respiratory tract.^[9]^ Evidence suggests that CRP is associated with the presence of potential bacterial pathogens in sputum.^[10]^ However, due to the small sample size of current studies, there is insufficient evidence for the clinical value of CRP in guiding the use of AECOPD antibiotics.

Therefore, the aim of this study was to evaluate the value of CRP-guided treatment strategies for AECOPD patients.

## 2 Methods

This study has been registered in PROSPERO (http://www.crd.york.ac.uk/PROSPERO), registration number: CRD42020165989. The procedure of this protocol is based on PRISMA-P guidance.

### 2.1 Database and search strategy

The following databases have been searched: 3 English medical databases (Cochrane Library, PubMed, and Embase) and 4 Chinese medical databases (China National Knowledge Infrastructure Database [CNKI], Chinese Biomedical Literature Database [CBM], VIP Chinese Science and Technology Periodical Database [VIP], and Wanfang Data). The databases are extensively searched from their inceptions up to May 11, 2020. The search strategy is based on the guidance of the Cochrane Handbook. Terms searched include: (Anti-Bacterial Agents OR Agents, Anti-Bacterial OR Anti-Bacterial Agents OR Antibacterial Agents OR Agents, Antibacterial OR Anti-Bacterial Compounds OR Anti-Bacterial Compounds OR Compounds, Anti-Bacterial OR Bacteriocidal Agents OR Agents, Bacteriocidal OR Bacteriocides OR Anti-Mycobacterial Agents OR Agents, Anti-Mycobacterial OR Anti Mycobacterial Agents OR Antimycobacterial Agents OR Agents, Antimycobacterial OR Antibiotics OR Antibiotic) AND (Pulmonary Disease, Chronic Obstructive OR COPD OR Chronic Obstructive Pulmonary Disease OR COAD OR Chronic Obstructive Airway Disease OR Chronic Obstructive Lung Disease OR Airflow Obstruction, Chronic OR Airflow Obstructions, Chronic OR Chronic Airflow Obstructions OR Chronic Airflow Obstruction) AND (C-Reactive Protein OR C Reactive Protein OR Protein, C-Reactive).

To guarantee comprehensive search, all relevant publications are researched, including academic dissertation and conference articles. The language is limited to Chinese and English.

### 2.2 Inclusion criteria

#### 2.2.1 Types of studies

Only human randomized controlled trials (RCTs) are included.

#### 2.2.2 Types of participants

Patients who had a diagnosis of COPD and were presenting with an acute exacerbation of COPD with at least one of the Anthonisen criteria.^[6]^

No restrictions regarding age, gender, condition duration or severity were applied. Diagnostic criteria refer to GOLD^[1]^.

#### 2.2.3 Types of intervention

The experimental group was tested CRP when antibiotics were administered or adjusted. Acceptable control groups include: the usual-care group and the GOLD strategy-guided group.

#### 2.2.4 Types of outcome measures

Primary outcomes: the antibiotic use and the Clinical COPD Questionnaire (CCQ). CCQ is a 10-item scale with a score ranging from 0 (very good) to 6 (extremely poor).

Secondary outcomes: EQ-5D utility scores; adverse events.

### 2.3 Exclusion criteria

(1) Non-RCTs should be excluded.
(2) Non-human should be excluded.
(3) Patients with pneumonia, asthma, bronchiectasis, other infection requiring antibiotic treatment, instable congestive heart failure, active intrathoracic malignancy, pulmonary embolism.
(4) The unrelated and duplicated documents have been deleted.
(5) No available data.

### 2.4 Data collection and extraction

Referring to the Cochrane collaborative network system evaluator handbook^[11]^: importing the search results into the document management software of EndNote (version:X9; Thomson Research Soft Company, USA); excluding the duplicate literature using EndNote X9 and excluding the unrelated articles by reading the title and abstract; and reading the full text and reserving clinical studies that meet the inclusion criteria. Two researchers (AX and WXW) extract the data independently using a self-developed data extraction form. The differences encountered in the process have been resolved by discussing with another team member (ZCT), to determine, by agreement, the final selection of studies.

Data extraction contents include: general information: research ID, author, title, publication status, report sources, and fund support; methodology information: design, number of arms, random sequence generation, allocation concealment, blinding, incomplete outcome data, selective reporting, sample size calculation, and baseline comparability; participant information: diagnostic criteria, inclusion criteria, exclusion criteria, setting, population, sample size, age, gender, and course of disease; intervention information: name of intervention and comparation, outcomes; and adverse events.

**Figure 1:**
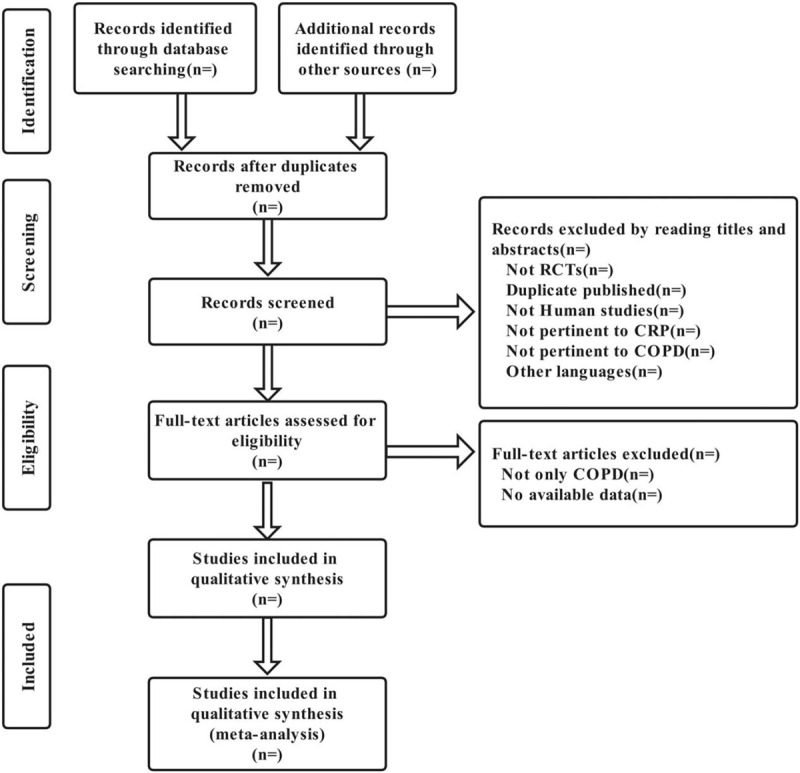
Flow chart of the selection process. COPD = Chronic obstructive pulmonary disease, CRP = C-reactive protein, RCTs = randomized controlled trials.

The selection process was showed in a PRISMA flow chart (http://www.prisma-statement.org/) (Fig. 1).

### 2.5 Assessment of methodologic quality

The Cochrane risk assessment tool has been used.^[12]^ Risk of bias has been assessed as follows: adequacy of generation of the allocation sequence, allocation concealment, double blinding, incomplete outcome date, selective outcome reporting, follow-up, and other bias. These domains classify “Yes” if adequate, “No” if not adequate, and “Unclear” if not well described by the authors in such a way that its adequacy is describable.

The 2 researchers (AX and WXW) independently assessed the risk of bias for each included study. “L,” “H,” and “U” have been used as a code for the evaluations of the above bias risks. “L” indicates a low risk of bias, “H” indicates a high risk of bias, and “U” indicates the risk of bias is unclear. Disagreements resolved by discussion between all the researchers. When necessary, the study authors have been contacted to inquire some missing information. Trials of high risk of bias will be considered when conducting sensitive analysis.

### 2.6 Data synthesis and analysis

Review Manager Software (RevMan, Version 5.3 for windows, The Cochrane Collaboration, Oxford, England) has been used to analyze and synthesize the outcomes. Clinical heterogeneity can be derived from the potential factors such as race, gender, control group characteristics. Quantitative synthesis has been done when clinical heterogeneity is not considered by at least 2 authors in discussion. Continuous variable has been described by mean difference (MD), *P* value, and 95% confidence interval (CI). For dichotomous outcomes, the relative risk (RR) has been used, with 95% CI and P-values, to evaluate the efficacy and safety of CRP testing guiding antibiotic prescribing. *I*^*2*^ test has been used to judge the heterogeneity of meta-analysis. *I*^*2*^ value > 50% or more will be considered as an indication of substantial heterogeneity. If heterogeneity exists in the pooled studies, the data have been analyzed using a random effects model. Otherwise, a fixed effect model has been adopted. If there is significant clinical heterogeneity, the cause of heterogeneity should be explored, and sensitivity analysis or subgroup analysis should be performed when necessary. Sensitivity analysis has been used to ensure the robustness of results by eliminating low-quality trials. Subgroup analysis will be performed according to the characteristics of the study subjects, such as different interventions, treatment duration, and outcome measures. If the data extraction is insufficient, qualitative analysis will be adopted.

### 2.7 Publication bias

The publication bias has been analyzed using funnel plot when the number of studies included in a meta-analysis is no < 10. If the number of included studies is < 10, the Egger test will be applied. The analysis software is R 3.5.1 for Windows.

### 2.8 Quality of evidence

This study evaluates the evidence according to GRADE standard, which refers grading of recommendations assessment, development and evaluation. These factors that may reduce the quality of evidence should be considered, such as limitations in study design, inconsistency of results, indirectness of evidence, inaccuracies, and publication bias. In addition, large magnitude effect, possible confounders that can reduce the effect and dose-response gradient that increase the quality of evidence cannot be ignored. GRADE Pro GDT online software will be used to form the summary of findings table (SoF table).

## 3 Discussion

AECOPD lead to increased morbidity, emergency hospital attendances, hospitalizations, health care costs, and more rapid disease progression and deterioration in quality of life. Antimicrobial therapy is necessary for the acute exacerbation of bacterial infection. Inappropriate use of antibiotics leads to waste of resources and bacterial resistance, and has a negative impact on the patient's microbiota.^[13]^

The use of biomarkers to identify bacterial infections in AECOPD has become a research hotspot in recent years. CRP is a sensitive biomarker for inflammation and tissue damage throughout the body.^[14]^ A study of patients with lower respiratory infections reported that CRP may help in clinical decision-making and guide the use of antibiotics.^[9]^ CRP levels are significantly higher during AECOPD compared to baseline levels, especially if a bacterial origin is likely.^[15]^ A randomized controlled trial involving acute exacerbations of COPD recruited from primary care clinics showed little difference in clinical cure rates with antibiotics or placebos in patients with CRP levels below 40 mg per liter.^[16]^ It is worth studying whether CRP can reduce the use of antibiotics without harming patients. At present, only a few studies evaluate the role of CRP in helping doctors to make antibiotic treatment in AECOPD patients.

Therefore, through systematic review and meta-analysis, we hope to effectively summarize the existing evidence, evaluate the clinical value and safety of CRP in guiding the use of AECOPD antibiotics, and provide a basis for clinical decision-making.

### 3.1 Ethics and dissemination

This review does not require ethical approval because the included studies are published data and do not involve the patients’ privacy. The results of this review will be reported in accordance with the PRISMA extension statement and disseminated to a peer-reviewed journal.

## Author contributions

**Conceptualization:** Xing An, Qingsong Huang.

**Data curation:** Xing An, Chuantao Zhang, Xiangwen Weng.

**Software:** Xiangwen Weng, Zhu Zeng.

**Supervision:** Wei Xiao, Zengtao Sun.

**Writing – original draft:** Xing An, Xiangwen Weng.

**Writing – review & editing:** Chuantao Zhang, Wei Xiao, Zengtao Sun, Zhu Zeng, Qingsong Huang.
